# Melatonin promotes the growth and development of lambs by increasing growth hormone and testosterone, targeting on apoptosis signaling pathway and intestinal microflora

**DOI:** 10.3389/fendo.2022.966120

**Published:** 2022-08-19

**Authors:** Wenkui Ma, Hao Wu, Guangdong Li, Laiqing Yan, Likai Wang, Mengmeng Zhao, Shengyu Guan, Shang Xu, Xiaokai Guo, Fenze Liu, Pengyun Ji, Abulizi Wusiman, Guoshi Liu

**Affiliations:** ^1^ National Engineering Laboratory for Animal Breeding, Key Laboratory of Animal Genetics and Breeding of the Ministry of Agricultural, Beijing Key Laboratory for Animal Genetic Improvement, College of Animal Science and Technology, China Agricultural University, Beijing, China; ^2^ Inner Mongolia Golden Grassland Ecological Technology Group Co., LTD., Inner Mongolia, China; ^3^ College of Animal Science, Xinjiang Agricultural University, Urumqi, China

**Keywords:** melatonin, lamb, muscle, fat, intestinal microbe, apoptosis

## Abstract

Melatonin is an indole-like neuroendocrine hormone. A large number of studies have shown that melatonin can improve production performance of ewes, but it is not clear in lambs. In this study, the growth and development of the 2-month-old lambs implanted with melatonin were monitored for 60 days. The results showed that the growth rate of body weight and body skew length of lambs with melatonin treatment were significantly improved compared to the controls. The similar results were also observed in red blood cell count, hematocrit, red blood cell volume distribution width, the levels of growth hormone, testosterone, immunoglobulin A, immunoglobulin M and albumin. In addition, the cross sectional area of muscle fibers and adipose cells of lambs with melatonin implantation were also significantly increased compared to the controls (*P*<0.05). To further explore the potential mechanisms, the muscle and adipose tissue were selected for transcriptome sequencing. KEGG enrichment results showed that melatonin regulated the expression of genes related to apoptotic signaling pathway in muscle and adipocytes. Since the intestinal microbiota are involved in the nutritional balance and animal growth, the 16SrRNA sequencing related to the intestinal microbiota was also performed. The data indicated that the structural differences of fecal microflora mainly occur in the pathways of Cardiovascular disease, Excretory system and Signaling molecules and interaction. In brief, melatonin promotes the growth and development of lambs. The potential mechanisms may be that melatonin increased the growth hormone and testosterone mediated apoptosis signaling pathway and regulated intestinal microbial flora. Our results provide valuable information for melatonin to improve the production of sheep husbandry in the future.

## Introduction

Melatonin is a tryptophan derivative, mainly secreted by the pineal gland of vertebrates at night and it is also widely distributed in bacteria and plants (1). Melatonin has a variety of functions in mammals, such as regulating biological rhythm, participating in immune regulation, inhibiting tumorigenesis, reducing oxidative stress and delaying aging (2).. Many physiological functions of melatonin are mediated primarily through the activation of receptors MT1 and MT2 (3). As to its functions in reproductive system, melatonin regulates seasonal reproductive activities of sheep such as modification of estrus cycle through the hypothalamic-pituitary-gonad axis (4). For example, *in vitro*, melatonin supplementation improves the maturation rate and cleavage rate of sheep oocytes by exerting its antioxidant function (5). *In vivo* melatonin subcutaneously injection during estrus increased the level of estradiol in the blood of ewe, and promoted embryo development as well as the pregnancy rate in the awes with natural mating or embryo transferring (6). Melatonin also improved the reproductive capacity of rams by modulation of testicular function (7). Evidence has been accumulated to show melatonin’s great application value in the sheep husbandry (8). The mechanisms of melatonin on mammalian physiology are multiples. These include its antioxidant and anti-inflammatory activities, energy metabolism, biorhythm regulation and mitochondrial biogenesis (9). Some of these activities are mediated by melatonin receptors and others are receptor-independent (10). In addition, some actions vary based on the dosages and delivery routes.

There are several melatonin delivery routes to improve the growth and reproductive performance of sheep, but a predominant route is subcutaneous implantation. By use of this method during cashmere production cycle, melatonin improved regeneration rate of skin secondary hair follicles and cashmere production (11). In the non-breeding season, melatonin regulated the estrus time of ewes and advances their estrus (12). Combined the controlled internal drug release devices and equine chorionic gonadotrophin (CIDR-ECG) with melatonin implantation in Anglo-Nubian goats significantly increased their luteal number, progesterone concentration, pregnancy rate and fertility (13). In the non-breeding season (at the beginning of May) melatonin implantation significantly improved the endocrine and exocrine function of testicles and scrotal circumference as well as quality parameters of the ejaculate in the Hungarian native breed Black Racka rams (14). Lamb weaning stress has always been an important issue which directly limits sheep production. As we know that melatonin implantation in pregnant ewes promoted fetal growth and reduced mortality of newborn lambs (15). However, the effects of melatonin on growth and development of lambs after weaning has not been investigated.

The growth and development of lambs involve in muscle and fat growth, immune activities and intestinal microflora regulation. Melatonin can regulate the release of inflammatory factors by interfering T cell differentiation and humoral and cellular immune activities (16). Melatonin can inhibit TLR4-mediated IL-6 expression in ewes choroid plexus (17). Melatonin is mainly synthesized in mitochondria (18). Mitochondria is the main site of energy metabolism (19). Melatonin maintains mitochondrial homeostasis and the ATP synthesis (20). Therefore, melatonin affects the metabolic activities of the body through its function on mitochondria. Evolutionally, mitochondria are the decedents of the bacteria of purple nonsulfur bacteria (R. rubrum) which have the capacity to synthesize melatonin (21). This principal is also applied to the intestinal microflora. Intestinal microflora not only affects nutrient absorption, immune regulation, but also energy metabolism in mammals (22, 23). Melatonin can modify the distribution and structures of intestinal microflora in suckling piglets (24). We hypothesize that the growth and development of lambs will also be influenced by their intestinal microflora which can be regulated with melatonin supplementation.

In the study, we will investigate whether melatonin administration will impact the growth and development of lambs. If it does, what are the potential mechanisms including the signal transduction pathways? Whether the crosstalk between melatonin and intestinal microbiota is involved in this process? To clarify these fundamental issues will provide valuable information not only for basic research but also for increasing the production in sheep husbandry.

## Materials and methods

### Experimental animals

The lambs of the Hu Sheep (Ovis aries) were from Golden Grassland Ecological Technology Co., LTD., Autonomous Region of Inner Mongolia. A total of 120 healthy 2-month-old male lambs with the body weight of about 19kg were selected. All the animal experimental design was in accordance with the regulations of animal Welfare Committee of China Agricultural University (No : AW01502202-1-2).

### Chemicals and regents

Follicle-Stimulating Hormone, FSH (B03PZB), Growth Hormone, GH (B12PZB), Luteinizing Hormone, LH(B04PZB), Androgens,T (B10PZB) and cortisol, COR (COR-D10PZB) were purchased from Beijing North Biotechnology Research Institute Co., LTD.; Estradiol, E2(B05PZB) was purchased from Tianjin Union Medical Science and Technology Co., LTD.; Superoxide dismutase, SOD ((A001-1-2), Malonaldehyde, MDA(A003-1-2), Glutathione peroxidase, GSH-PX (A005-1-2), Catalase, CAT (A007-1-1), Immunoglobulin m, IGM (Jh-00015), Immunoglobulin g, IGG (Jh-00013) and Immunoglobulin a, IGA(Jh-00014) were purchased from Nanjing Jiancheng Biological Engineering Research Institute Co., LTD.; Glutamic-pyruvic transaminase, ALT (Alanine substrate method, B2008),Glutamic oxalacetic transaminase, AST (aspartic acid substrate method, B2009), Total protein, TP (biuret method, B2010), Albumin, ALB (bromocresol green method, B2011), alkaline phosphatase, ALP (NPP substrate-AMP buffer method, B2014), Urea nitrogen, UREA(Urease-glutamate dehydrogenase method, B2017), Serum creatinine, CRE (Creatine oxidase method, B2018), Uric acid, UA (uricase method, B2019), Glucose, GLU (Glucose oxidase method,. B2007) were purchased from Beijing Beijian•xinchuangyuan Biotechnology Co., LTD.

### Experimental design

Lambs were randomly divided into 3 groups which was with 40 lambs for each group. Melatonin was implanted subcutaneously in the neck (Institute of Special Products, Chinese Academy of Agricultural Sciences, No: 2019/4/18) with 0mg, 3mg/kg and 4.5mg/kg, respectively for each lamb in each group. The lambs were fed with the total mixed diet at 9:00 and 17:00, but accessed to the water freely. The ratio of concentrate to coarse feed was 1:3. The basic components of concentrate feed include corn,soybean meal,corn dry distiller’s grains,calcium carbonate,vitamins and trace elements. Nutrients include crude protein ≥18%, crude fiber ≥15%, ash ≤10%, calcium 0.5%-20%, total phosphorus 0.3%, sodium chloride 0.3%-20%, moisture ≤13%, lysine ≥0.5%. The body height, oblique length, chest circumference, chest width, chest depth, anterior duct and posterior duct were measured morning on day 0d, 30d and 60d after an overnight fasting.

### Routine blood test

5mL blood was collected from the jugular vein of the lambs, on 60d of the study. The samples were stored in EDTA tubes and transported at 4°C. Blood routine tests were performed with blood cell analyzer (Shenzhen Mindray Bio-medical Electronics Co., LTD., BC2800Vet, Shenzhen, China).

### Detection of hormones and physiological and biochemical parameters

5mL blood was collected from the jugular vein of the lambs on day 0d, 30d and 60d. The samples were kept at room temperature for 2h, centrifuged at 3000r/min for 8min, and the serum was stored at -20°C. All samples were tested according to the instructions of the kit as above 1.1. Chemicals and regents.

(1) Reproductive hormones [FSH, GH, LH, T, E2] and Antioxidant factors [SOD, MDA, GSH-PX, CAT, COR] were detected by radioimmunoassay (Xi’an Nuclear Instrument Factory, XH6080; Xian, China) in Beijing North Biotechnology Research Institute Co., LTD.

(2) Physiological and biochemical parameters: ALT, TP, ALP, UREA, CRE, UA, GLU were measured following the manufacturer’s instructions.

(3) Immune factors: IGM, IGG, IGA were detected by 721 spectrophotometer and colorimetry.

(4) Melatonin: The serum samples were mixed with methanol in a ratio of 1:4 and then oscillated in a vortex. After centrifugation (12000 r/min for 10 min), the supernatant was collected and filtered into the membrane for use. Melatonin detection was performed using high performance liquid mass spectrometry (AGilent1290-G6470, Santa Clara, CA, USA) at the Central Laboratory of The Institute of Animal Science, Chinese Academy of Agricultural Sciences (Beijing, China).

### Tissue staining

The sheep were stabilized, the surface of the longissimus muscle of the back was sheared, disinfected with iodophor, and then deiodized with 75% alcohol. Using a scalpel, slice the sterilized skin and fat in turn, using forceps and direct scissors to obtain the longest muscle and fat in the back. The longissimus muscle and back adipose tissue were fixed with 4% paraformaldehyde (Beijing Regen Biotechnology Co., LTD., No. DF0135) for 24h, then the tissue and label were placed in the dehydration box. The dehydration box is put into the dehydrator (Wuhan Junjie Electronics Co., LTD., JJ-12J, Wuhan, China) and then the gradient alcohol (Sinopagic Chemical Reagent Co., LTD., 100092683) is used for dehydration, and then the tissue is dipped in wax. The wax-soaked tissue is embedded in the embedding machine (Wuhan Junjie Electronics Co., LTD., JB-P5, Wuhan, China). The trimmed wax blocks were cooled at -20°C (Wuhan Junjie Electronics Co., LTD., JB-P5, Wuhan, China) and sliced in a paraffin slicer (Shanghai Leica Instrument Co., LTD., RM2016, Shanghai, China) with a thickness of 4μm. The slices were floated in the spreading machine (Jinhua Kedi Instrument Equipment Co., LTD., KD-P, Zhejiang, China) and the tissues were flatten in water at 40°C. The slides (Servicebio, G6004) were picked up and the slices were baked in the oven at 60°C (Tianjin Laiborey Instrument Equipment Co., LTD., GFL-230, Tianjin, China). Water drying wax baking out of room temperature storage for later. The slices were then placed in xylene I (Sinopagic Chemical Reagent Co., LTD., 10023418) for 20min, xylene II for 20min, anhydrous ethanol I for 5min, anhydrous ethanol II for 5min, 75% alcohol for 5min, and washed with water. The slices were stained with hematoxane dye (HE dye kit purchased from Servicebio, G1003, Wuhan, China) and sealed with neutral gum (Sinopharming Chemical Reagents Co., LTD., 10004160). Microscopic examination and image analysis were performed using Nikon Eclipse E100 (Tokyo, Japan).

### RNA-seq and qPCR

Total RNA was extracted from muscle and adipose tissue by TRIzol (Ambion,15596026), and genomic DNA was removed by DNase I (TaKara). The quality of RNA samples was detected by 2100 Bioanalyser (Agilent) and ND-2000(NanoDrop Technologies). After the samples were qualified (OD260/280 = 1.8-2.2, OD260/230≥2.0, RIN≥6.5), 28S:18S≥1.0, >2μg) were sequenced.

TruSeqTM RNA Sample Preparation Kit (Illumina, San Diego, CA) was used for the construction of RNA library. SuperScript double-stranded cDNA synthesis Kit (Invitrogen, CA) was used to inversely synthesize cDNA and form a stable double-stranded structure. After cDNA enrichment by PCR (sample Preparation Kit (Illumina, San Diego, CA) Kit), DNA clean Beads (DNA Clean Beads) are screened for 200-300 bp bands. After quantified by TBS380 (Picogreen), Illumina HiSeq XTEN/NovaSeq 6000 sequencing platform was used for high-throughput sequencing with a read length of PE150.

QPCR procedure: Incubation at 95°C for 3 min (activation of FastStart DNA polymerase), 95°C for 10 seconds, 60°C for 30 seconds. 39 cycles were obtained with appropriate extension time for a single fluorescence acquisition. FAS, P53, GADD45B, PARP1, CytC, BAx, Bcl-2, APAF1, FAS-L, TNF-α, IL1B, Bcl-XL, ATM, IL3, CASP3, CASP8 were selected for verification, and GAPDH was used as control. All gene validation was repeated for 3 times, and gene expression was evaluated by 2-^△△^CT method. The primers were verified as shown in **Table 1**. Additional Requirements

**Table 1:** Primer sequence.

Primer	sequence (5’-3’)	Product size(bp)		Tm (°C)
GAPDH	F: GTCGGAGTGAACGGATTTGG	97		60
	R: TGAAGGGGTCATTGATGGCA			
FAS	F: TGCACACCAACTAGCAACAC	188		60
	R: AGTTGCCTCCCTTCATCACTT		
P53	F: AAAAGTCCAGAGCCACCATCC	120		60
	R: TGTGGCCGCCGAAGC		
GADD45B	F: ACCTGTCGTGAGGCTGTTTT	101		60
	R: TCACCGTCTGCATCTTTTGC			
PARP1	F: TGATGTTGAGGTGGAGGGGT	102		60
	R: CCTTTGCCTGAAACATCTGTCG		
CytC	F: ATGCTGCCAAGAATGTCGTC	136		60
	R: TTGCCTCGCCATTTGAAAGC		
Bax	F: GACGGCAACTTCAACTGGG	227		60
	R: ACAAAGATGGTCACGGTCTGC		
BCL-2	F: GCCCTGTGGATGACCGAGTA	128		60
	R: GACAGCCAGGAGAAATCAAACA		
APAF1	F: TGTGCATTGGGTGTCAGTTG	75		60
	R: AATCGTGCGCAAAGGTTCTG		
FAS-L	F: TTAACAGGCAAGCCCAACTC	98		60
	R: AGGCTGCCCTTCTTGTACTTC
TNF-α	F: TAACAAGCCGGTAGCCCACGTT	109		60
	R: GTCTTTCAGCTCCACGCCGTT
IL1B	F: AATATGGAAAAGCGATTCGTCT	141		60
	R: GCCACCTCTAAAACGTCCCA
BCL-XL	F: ACTTGGATGGCTACTTACCTG	83		60
	R: TAGAGTTCCACAAACGTGTCC
ATM	F: TGTTCATCTTATCACCGTGACC	170		60
	R: TTCCCCTCCTTTGTTAAGTGC
IL3	F: AGCGTTCATGACATTTGCCACA	118		60
	R:AATAAGAATTGAATCTTCCGTGGGTGT
CASP3	F: AACGATCTTACACGGAAGCAA	131		60
	R: CCATGCCAGTATTTTCGTGGA
CASP8	F: ACATCCGACACAGTTTACCG	90		60
	R: CTGCTCCCGTGCTATGCT

### 16S intestinal microbiome sequencing

Rectal feces were collected in a sterile environment, frozen with liquid nitrogen and stored at -80°C. Stool microbiome DNA extraction method refer to PowerSoil DNA Isolation Kit (MoBio Laboratories, Carlsbad, CA) [Omega DNA Kit]. The samples are stored at -20°C after passing quality inspection. Primers F (5’-ACTCCTACGGGAGgCAGCAGcag-3’) and R (5’-GGACTACNNGGG TATctaat-3’) were used to amplify V3-V4 region of bacterial 16SrRNA gene. PCR products were used to construct microbial diversity sequencing library. Illumina Miseq PE300 high-throughput sequencing platform was used for Paired end sequencing in Beijing Alvesen Gene Technology Co., LTD. QIIME1 (V1.8.0) software was used to split the samples according to Barcode sequence, and Pear (V0.9.6) software was used to filter and splice the data. The uPARSE algorithm of Vsearch (V2.7.1) software was used to perform OTU clustering (Operational Taxonomic Units) for high-quality sequences, and the similarity threshold was 97%. The RDP Classifier algorithm was used for comparison with Silva128 database, and the 70% confidence threshold was set to obtain the species classification information corresponding to each OTU. Venn plots, rank-abundance curves and species composition histogram were analyzed using R (V3.6.0) software based on species annotation and relative Abundance results. R (V3.6.0) software was used for PLS-DA analysis and partial least square discriminant analysis. QIIME1 (V1.8.0) was used to calculate the beta diversity distance matrix, R (V3.6.0) software was used for clustering heat map analysis based on Weighted Unifrace distance, and Phython (V2.7) software was used for LEfSe analysis.

### Statistical analysis

The data were sorted by Excel. The results were expressed as mean ± SEM. One-way ANOVA was used to analyze the normality of the samples and followed by Duncan test to determine the statistically significant difference between the groups with the help of SPSS 26.0(IBM SPSS Statistics, Armonk, NY, USA). *P*<0.05 was taken as the criterion of significance of difference.

## Results

### Effects of melatonin on growth performance of lambs

**Figure 1:**
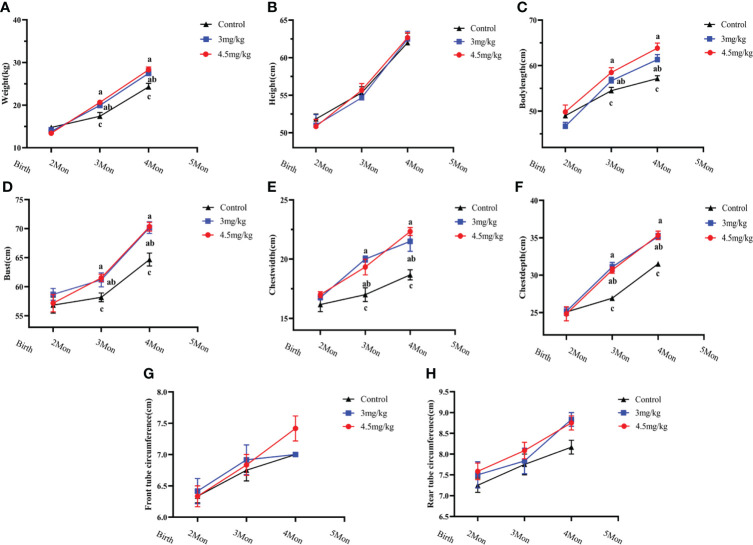
Effects of melatonin on growth performance of lambs. **(A)** body weight; **(B)** body height; **(C)** body oblique length; **(D)** chest circumference; **(E)** chest width; **(F)** chest width; **(G)** anterior tube circumference; **(H)** posterior tube circumference Different letters indicate *P*<0.05 vs control.

The body weight (**Figure 1A**), body oblique length (**Figure 1C**), chest circumference (**Figure 1D**), chest width (**Figure 1E**) and chest depth (**Figure 1F**) in melatonin implanted group were significantly greater than those in the control group (*P*<0.05) with the better results in 4.5 than 3 mg/kg melatonin treated group. There were no significant differences in body height (**Figure 1B**), anterior canal circumference (**Figure 1G**) and posterior canal circumference (**Figure 1H**) among all groups (*P*>0.05).

### Effects of melatonin on the parameters of physiology and biochemistry in lambs

After 30 days of melatonin implantation, the albumin content of lambs increased gradually and was significantly higher than that of control group (*P*<0.05). No significant differences on globulin and white bulb ratio were detected among the groups (*P*>0.05) (**Table 2**). Interestingly, the RBC count, hematocrit and RBC volume distribution width were significantly higher in lambs with 4.5mg/kg melatonin implantation than those in the 3mg/kg group (*P*<0.05) while these differences were not observed between the lambs with melatonin implantation and controls (*P*>0.05) (**Table 2**). WBC count, mean RBC volume, mean RBC hemoglobin content, mean PLATELET volume, platelet distribution width and platelet hematocrit had no significant differences among all groups (*P*>0.05) (**Table 3**) and these were the case on levels of glucose, urea and uric acid among groups (*P*>0.05) (**Table 4**).

**Table 2:** Effects of melatonin implantation on liver function of sheep.

Type	Time	Group		
		Control	3mg/kg	4.5mg/kg
	0d	11.67 ± 1.58	11.83 ± 2.12	13.33 ± 1.99
ALT	30d	8.17 ± 1.92^b^	9.50 ± 1.67^ab^	9.67 ± 1.36^a^
	60d	12.00 ± 1.71	15.83 ± 1.08	18.33 ± 2.99
	0d	33.10 ± 2.17	31.55 ± 1.95	31.40 ± 1.81
ALB	30d	28.85 ± 1.88^b^	31.52 ± 1.15^ab^	33.90 ± 1.46^a^
	60d	34.15 ± 0.68^B^	35.97 ± 0.51^AB^	37.15 ± 0.88^A^
	0d	21.03 ± 2.63	20.35 ± 1.45	18.87 ± 1.97
GLB	30d	25.95 ± 1.91	23.77 ± 0.74	26.20 ± 1.69
	60d	30.20 ± 2.03	27.97 ± 1.33	29.85 ± 0.40
	0d	1.66 ± 0.22	1.61 ± 0.17	1.79 ± 0.17
A/C	30d	1.16 ± 0.10	1.33 ± 0.06	1.29 ± 0.09
	60d	1.15 ± 0.08	1.30 ± 0.05	1.25 ± 0.04

glutamic-pyruvic transaminase, ALT; albumin, ALB; globulin, GLB; albumin/globulin, A/C. Different uppercase letters in the same row indicate P<0.01 vs control or same group in different treatment days, different lowercase letters indicate P<0.05 vs control or same group in different treatment days.

**Table 3:** Effects of melatonin implantation on blood routine indexes of the sheep.

Type	Time	Group		
		Control	3 mg/kg	4.5 mg/kg
WBC	60d	9.30 ± 0.84	9.25 ± 0.74	9.28 ± 1.34
RBC	60d	13.98 ± 0.29^a^	11.87 ± 0.35^b^	13.80 ± 0.83^a^
HCT	60d	36.12 ± 0.61^a^	32.82 ± 1.30^b^	35.32 ± 0.60^ab^
MCV	60d	25.90 ± 0.42	27.68 ± 0.61	27.00 ± 0.84
MCH	60d	8.72 ± 0.14	9.18 ± 0.28	8.87 ± 0.16
RDW	60d	19.85 ± 0.52^a^	18.17 ± 0.36^b^	19.07 ± 0.46^ab^
MPV	60d	3.82 ± 0.03	3.73 ± 0.06	3.87 ± 0.06
PDW	60d	14.92 ± 0.04	15.08 ± 0.11	14.93 ± 0.04
PCT	60d	0.27 ± 0.02	0.20 ± 0.05	0.25 ± 0.02

White Blood Cells, WBC; Red Blood Corpuscle, RBC; hematocrit, HCT. mean corpuscular volume, MCV; mean corpuscular hemoglobin concentration MCH; Red cell distribution width, RDW; Mean Platelet Volume, MPV; Platelet distribution width, PDW; Platelet cubic measure distributing width, PCT.

**Table 4:** Effects of implanted melatonin on sheep kidney function.

Type	Time	Group		
		Control	3 mg/kg	4.5 mg/kg
	0d	3.36 ± 0.39	3.43 ± 0.28	3.49 ± 0.21
GLU	30d	1.26 ± 0.33	1.56 ± 0.16	1.54 ± 0.17
	60d	5.83 ± 0.34	5.53 ± 0.37	6.43 ± 0.48
	0d	4.60 ± 0.40	3.97 ± 0.34	4.22 ± 0.35
UREA	30d	6.07 ± 0.61	6.65 ± 0.87	6.23 ± 0.63
	60d	5.83 ± 0.34	5.53 ± 0.37	6.43 ± 0.48
	0d	6.50 ± 0.43	5.5 ± 1.28	5.67 ± 0.67
UA	30d	7.17 ± 0.48	8.00 ± 1.41	9.00 ± 1.97
	60d	4.00 ± 0.97	5.33 ± 1.02	3.83 ± 0.91

glucose, GLU; urea nitrogen, UREA; Uric Acid, UA. Different uppercase letters in the data of the same row indicate highly significant differences (P < 0.01), different lowercase letters indicate P < 0.05 vs control.

### Effects of melatonin on reproductive hormones in lambs

**Figure 2:**
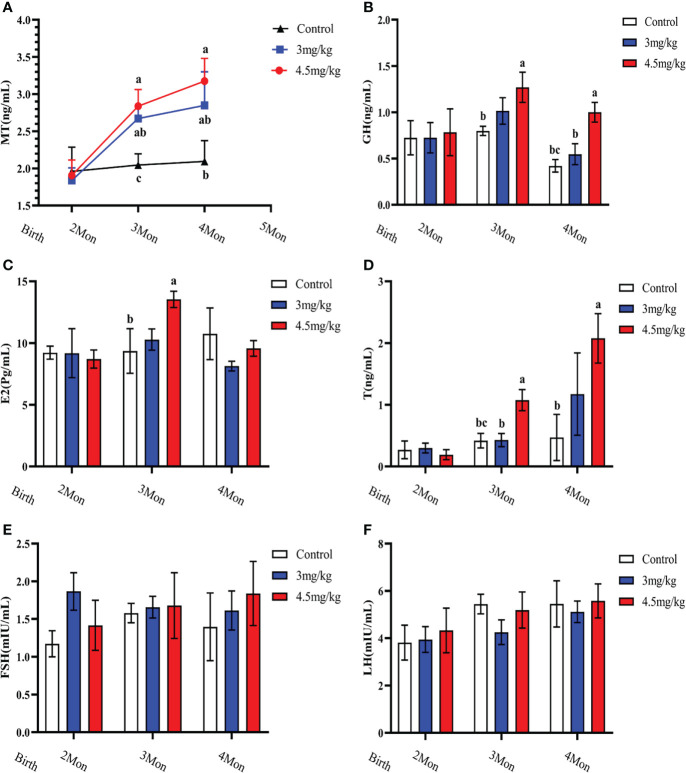
Effects of melatonin on blood melatonin levels and reproductive hormones in lambs. **(A)** Melatonin levels; **(B)** Growth hormone; **(C)** Estradiol; **(D)** Androgens; **(E)** Follicle-stimulating hormone; **(F)** Luteinizing hormone-releasing hormone. Note: Different lowercase letters indicate *P*<0.05 vs control.

Melatonin implantation significantly increased the blood melatonin levels of the lambs compared to the controls (*P*<0.05) (**Figure 2A**). The growth hormone (**Figure 2B**), estradiol (**Figure 2C**) and androgen (**Figure 2D**) in lambs with 4.5mg/kg of melatonin implantation were significantly increased compared to the controls (*P*<0.05). After 1 month melatonin implantation, androgen level in 4.5mg/kg group was significantly higher than that of 3mg/kg group (*P*<0.05) and at 2 months, the growth hormone level had the similar change as the androgen (*P*<0.05). The levels of luteinizing hormone releasing hormone (**Figure 2E**) and follicle-stimulating hormone releasing hormone (**Figure 2F**) had no significant difference among groups (*P*>0.05).

### Effects of melatonin on antioxidant and immune factors in lambs

**Figure 3:**
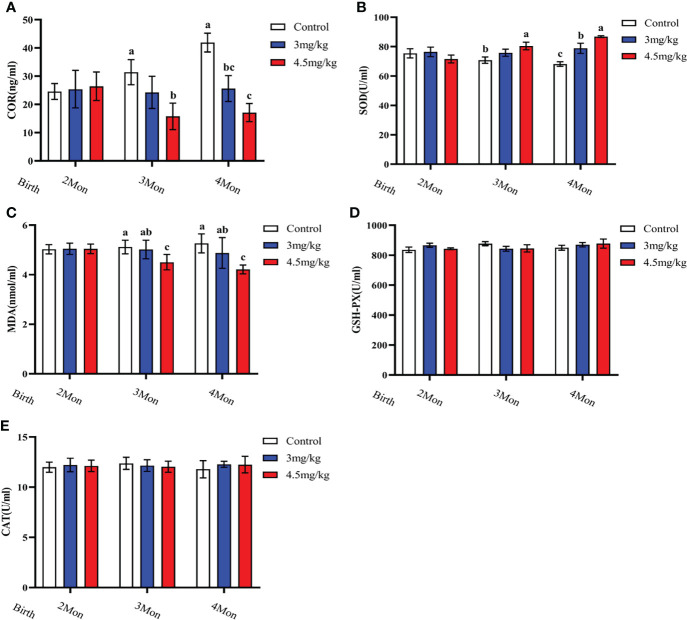
Effects of melatonin on antioxidant factors of lambs. **(A)** cortisol; **(B)** superoxide dismutase; **(C)** malondialdehyde; **(D)** glutathione peroxidase; **(E)** catalase. Different lowercase letters indicate *P*<0.05, and the same lowercase letters indicate *P*>0.05 vs contrpl.

**Figure 4:**
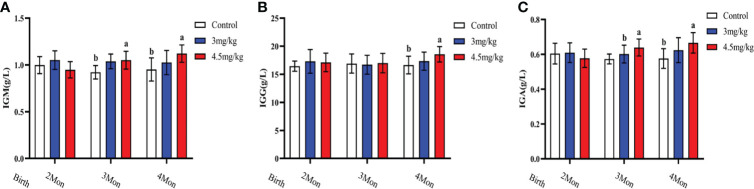
Effects of melatonin on immunoglobulins of lambs. **(A)** IgA; **(B)** IgG; **(C)** IgM Different lowercase letters indicate significant differences (*P*<0.05), and the same lowercase letters indicate insignificant differences (*P*>0.05).

Cortisol (**Figure 3A**) and malondialdehyde (**Figure 3C**) were significantly lower and superoxide dismutase (**Figure 3B**) was significantly higher in lambs with 4.5mg/kg melatonin implantation than those in other groups (*P*<0.05). Glutathione peroxidase (**Figure 3D**) and catalase (**Figure 3E**) were not significantly differences among groups (*P*>0.05). The levels of immunoglobulin A (**Figure 4A**) and immunoglobulin M (**Figure 4C**) in lambs with 4.5mg/kg melatonin implantation were significantly higher than those in other groups (*P*<0.05). But, there was no significant difference in the immunogiobulin G (**Figure 4B**) among the groups.

### Effects of melatonin on longissimus muscle and back adipose tissue in lambs

**Figure 5:**
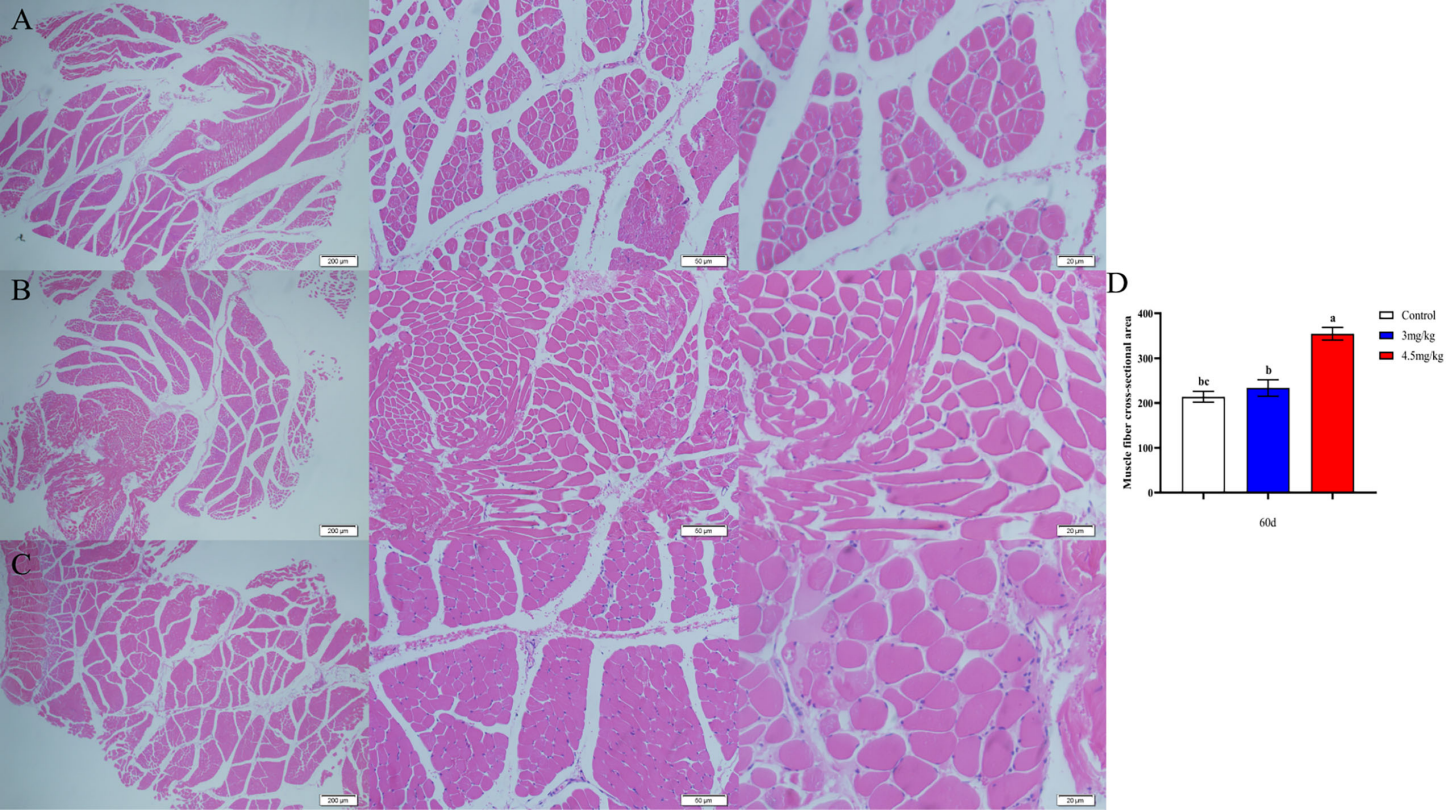
Cross-sectional muscle fiber diagram of the back muscles. **(A)** The cross section of the longissimus muscle in control group, 200×, 50×, and 20×, respectively; **(B)** The transverse section of the longissimus muscle in 3mg/kg group, 200×, 50×, and 20×, respectively; **(C)** The cross section of the longissimus muscle in 4.5mg/kg group, 200×, 50×, and 20×, respectively; **(D)** Statistical analysis of the cross-sectional area of the longissimus dorsi. Different lowercase letters indicate *P*<0.05 vs control.

**Figure 6:**
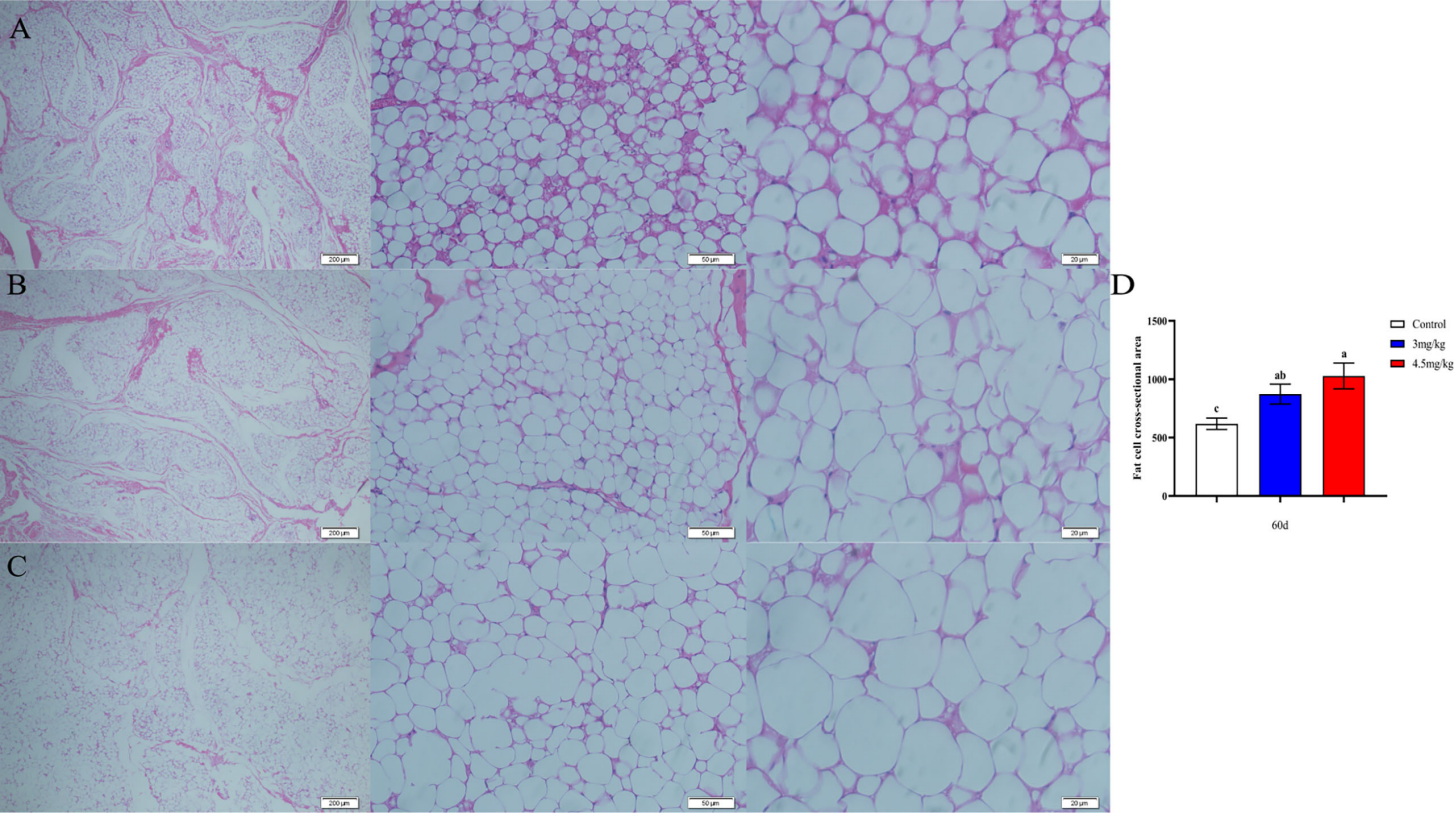
Cross-sectional cytogram of dorsal adipose tissue. **(A)** Transverse sections of the back adipose tissue in the control group, 200×, 50×, and 20×, respectively; **(B)** Transverse sections of the back adipose tissue in the 3mg/kg group, 200×, 50×, and 20×, respectively; **(C)** The cross section of the back adipose tissue in the 4.5mg/kg group, 200×, 50×, and 20×, respectively; **(D)** Statistical analysis of the cross-sectional area of dorsal adipose tissue. Different lowercase letters indicate *P*<0.05 vs control.

The cross-sectional area of longismus muscle tissue was significantly larger (**Figure 5C**) in lambs with 90 mg melatonin implantation than that in the other groups (**Figures 5A**, **B**) (*P*<0.05). The cross-sectional area of adipose tissue implanted with 3mg/kg (**Figures 6B**) and 4.5mg/kg (**Figures 6C**) melatonin was significantly higher than that of the control group (**Figures 6A**). The cross-sectional areas of adipose tissue were significantly larger in lamb with 60 and 90 mg melatonin implantations than that of the control group (**Figures 5D**, **6D**).

### Transcriptome analysis of back longissimus muscle and adipose tissue of lambs

**Figure 7:**
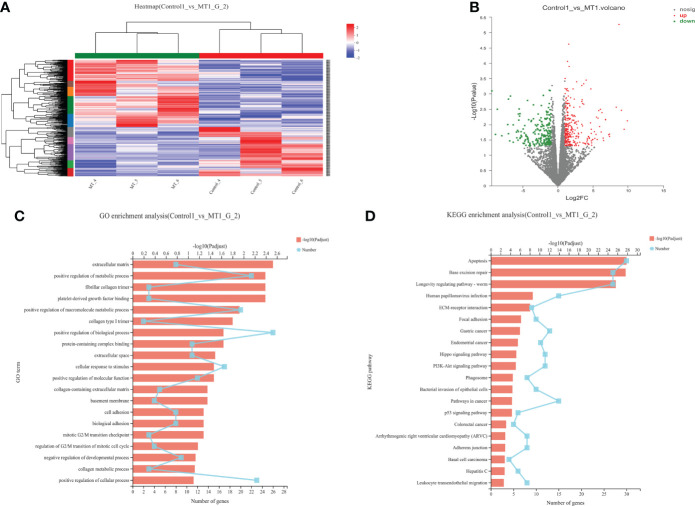
Transcriptome sequencing of back muscle tissue. **(A)** Cluster analysis; **(B)** Expression difference analysis; **(C)** GO enrichment analysis; **(D)** KEGG enrichment analysis.

**Figure 8:**
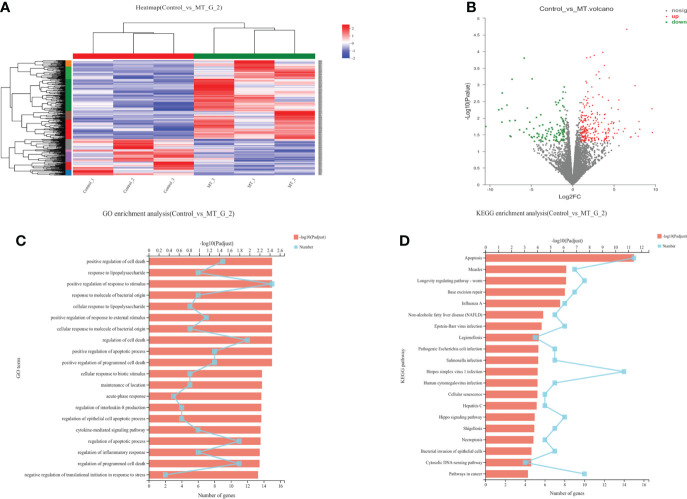
Transcriptome sequencing of back muscle tissue. **(A)** Cluster analysis; **(B)** Expression difference analysis; **(C)** GO enrichment analysis; **(D)** KEGG enrichment analysis.

**Figure 9:**
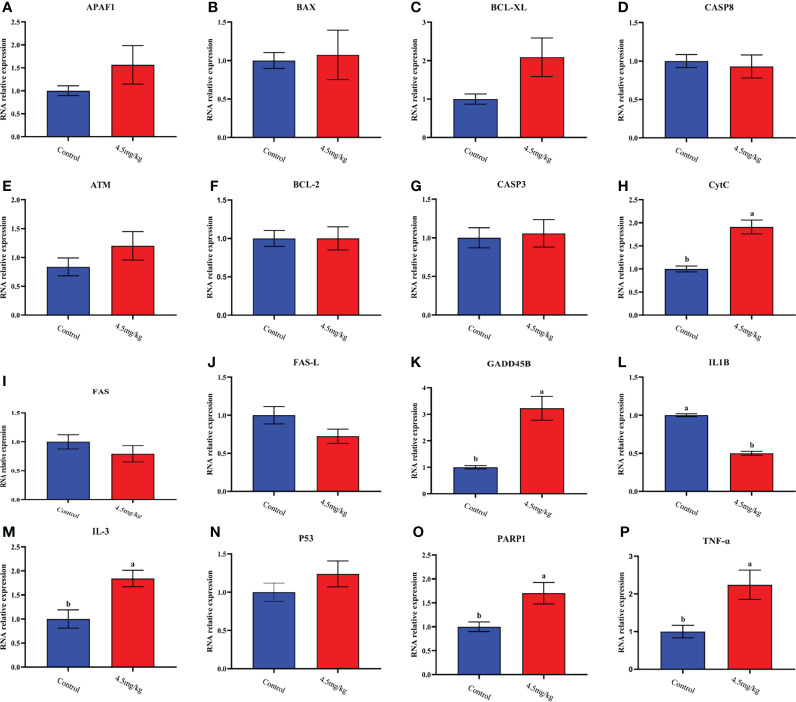
Quantitative analysis of apoptosis and collateral genes in back muscle tissue **(A)** APAF1; **(B)** BAX; **(C)** BCL-XL; **(D)** CASP8; **(E)** ATM; **(F)** BCL-2; **(G)** CASP3; **(H)** CytC; **(I)** FAS; **(J)** FAS-L; **(K)** GADD45B; **(L)** IL-1β; **(M)** IL-3; **(N)** P53; **(O)** PARP1; **(P)** TNF-ɑ; Different lowercase letters indicate *P*<0.05 vs control.

**Figure 10:**
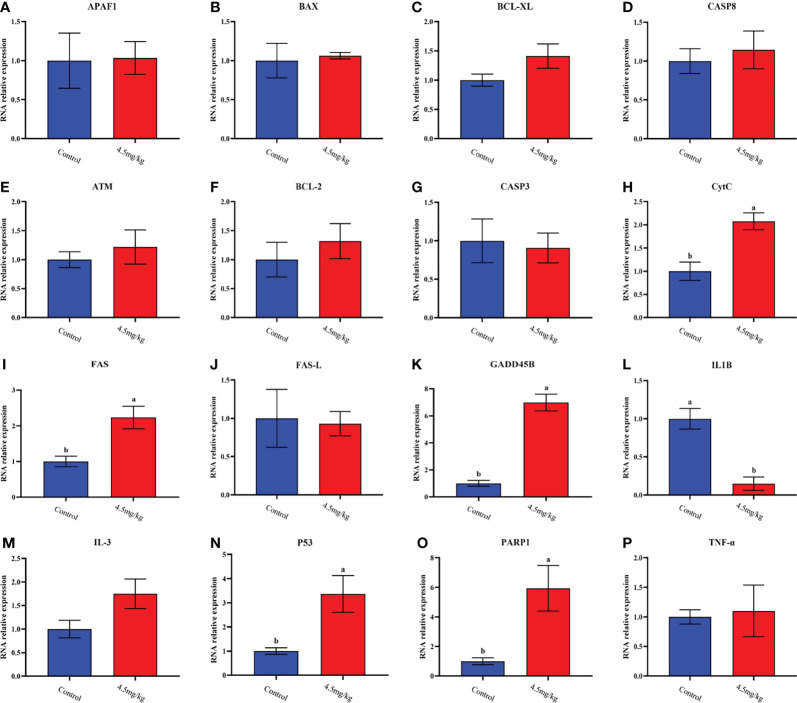
Quantitative analysis of apoptosis dredging genes in dorsal adipose tissue. **(A)** APAF1; **(B)** BAX; **(C)** BCL-XL; **(D)** CASP8; **(E)** ATM; **(F)** BCL-2; **(G)** CASP3; **(H)** CytC;**(I)** FAS; **(J)** FAS-L; **(K)** GADD45B; **(L)** IL-1β; **(M)** IL-3; **(N)** P53; **(O)** PARP1; **(P)** TNF-ɑ; Different lowercase letters indicate *P*<0.05 vs control.

The transcriptome analyses were performed from the back longissimus muscles and adipose tissues of lambs with 4.5mg/kg melatonin implantation and controls. The significant differences in gene expressions in back longissimus muscle and adipose tissue have been observed between the melatonin and the control groups (**Figures 7A**, **8A**). A total of 346 differentially expressed genes were detected in muscle tissue, of which 237 were up and 109 were down-regulated (**Figure 7B**). A total of 492 differentially expressed genes were detected in adipose tissue, of which 284 were up and 208 were down-regulated (**Figure 8B**). GO enrichment analysis showed that the differentially expressed genes in muscle tissue were mainly enriched in extracellular matrix and positive regulation of metabolic process pathway (**Figure 7C**). KEGG enrichment analysis showed that these differentially expressed genes were mainly enriched in Apoptosis and Base excision repair pathway (**Figure 7D**). GO enrichment analysis showed that the differentially expressed genes in adipose tissue were mainly enriched in positive regulation of cell death and response to lipopolysaccharide pathway (**Figure 8C**). KEGG enrichment analysis showed that these genes were mainly enriched in Apoptosis and Measles pathways (**Figure 8D**). Based on the KEGG enrichment analysis, differentially expressed genes in muscle and adipose tissues both were convergently enriched in the Apoptosis pathway. Therefore, sixteen genes in Apoptosis pathway were selected for validation. The expression levels of CytC, GADD45B, IL-3, PARP1 and TNF-ɑ in muscles were significantly higher and IL1-β were significantly lower in lambs with melatonin implantation than those in the controls (**Figure 9**). The expression levels of CytC, FAS, P53 and PARP1 in adipose tissue were significantly higher and IL1-β was significantly lower in lambs with melatonin implantation than those in controls (**Figure 10**). The expression of CytC, PARP1 and IL1-β genes in both muscle tissue and adipose tissue showed the similar changes after melatonin intervention in the Apoptosis pathway.,

### Effects of melatonin on intestinal microflora of lambs

**Figure 11:**
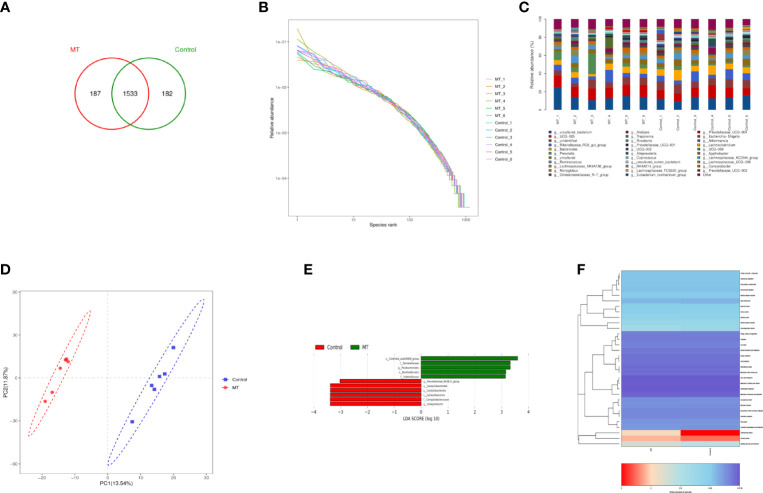
Sequencing analysis of gut microbiota. **(A)** Venn diagram; **(B)** Rank abundance diagram; **(C)** Histogram of genus-level species composition analysis; **(D)** OTU-based partial least squares discriminant analysis; **(E)** LDA distribution of LEfSe analysis based on taxonomic information Histogram; Species with differences in LDA score greater than 3, species with significant differences in abundance in different groups, the length of the histogram represents the impact size of significantly different species; **(F)** PICRUSt predicted differential pathway heat map A.

In order to explore the effect of melatonin on the intestinal microflora structure in the lambs, 6 lambs with 4.5mg/kg melatonin implantation and 6 lambs as untreated controls were selected for 16SrDNA sequencing, respectively. A total of 1902 Operational Taxonomic Units (OTUs) were identified by sequencing, among which 369 differences OTUs (**Figure 11A**) were identified. The values of Rank curve decreased steadily, indicating the good species uniformity and richness (**Figure 11B**). The analysis of species composition showed that there was no significant difference in the structure of microflora at the genus level among samples. But the proportion of different microflora among individuals was different (**Figure 11C**). Partial Least Squares Discrimination Analysis (PLS-DA) showed the significant differences of microbial composition between the melatonin treated lambs and the controls, the PC1 and PC2 were 13.54% and 11.87%, respectively (**Figure 11D**). LDA Effect Size analysis was used to evaluate the structure of microbial flora. When LDA score was set up as 3, melatonin treated and control groups were divided into 5 and 6 different microflora (**Figure 11E**) respectively, among them o_Clostridia_vadiaBB60_group had the most obvious influence. In addition, PICRUSt was used to analyze microbial community function. Based on the data of KEGG analysis, the differences in structural and functional pathways of fecal microflora are mainly concentrated in the pathways of Cardiovascular disease, Excretory system and Signaling molecules and interaction (**Figure 11F**). Melatonin treatment may accelerate the rate of Cardiovascular and cerebrovascular metabolic cycle. Therefore, the differences in Cardiovascular disease pathway are the most obvious.

## Discussion

Sheep is one of the most important species in animal husbandry, and mutton is one of the main meats in human diet. With the rapid development of social economy, sheep farms and associated industry are upsized to extremely large-scale. Traditional feeding methods no longer meet the demand of consumer for high-quality mutton. To produce high-quality mutton at low cost and meet consumer’s increased demand are huge challenge for scientists. Melatonin is a naturally occurring molecule present in almost all organisms with a variety of biological functions (25). A large number of studies have shown that melatonin can improve the efficiency of sheep reproductive performance (26, 27). The growth retardation of lambs after weaning is a frequently faced problem in large-scale breeding farms. To explore this problem, 120 post-weaning lambs were selected in this study to evaluate whether melatonin implantation will impact the growth retardation of lambs related to the weaning. Based on the pilot study, to achieve the obvious effects on the growth and development of lambs required around 60 days after melatonin implantation. Thus the 60 days or experimental period was selected.

The results showed that the body weight, body length, chest circumference, chest width and chest depth of lambs with melatonin implantation were significantly increased compared to the controls, and the effect was better in the 4.5mg/kg than that in 3mg/kg group. Many previous studies have reported the methods to improve growth and development of lambs. However, majority of these methods focused on the feed additives and antibiotics (28, 29). For example, dietary supplementation of oregano and cobalt mixture significantly improved growth performance, feed conversion, carcass weight, slaughter rate and immunity of lambs (30). However, these xenobiotics may have some unfavorable metabolic consequences for human body (31). In current study, melatonin was selected since melatonin is a naturally occurring molecule found in human body and with minimal side effects (32). Compared with other synthetic growth promoting products, melatonin has huge safety margin to animals and humans. In addition, previous studies have reported the regulatory effects of melatonin on sheep reproductive activities (33). Here, we reported that melatonin implantation at a certain dosage significantly improved the growth performance of lambs.

Firstly, we evaluated the safety parameters of the different dosages of melatonin on lambs. The results showed that alanine aminotransferase and albumin in 4.5mg/kg group were significantly higher than those in control group (*P*<0.05), but there were no significant differences in glucose, urea and uric acid level compared to the control (*P*>0.05). When we look at physiological markers in the blood, the results are variable in different melatonin dose groups. The RBC count, hematocrit and RBC distribution width in 4.5mg/kg group were significantly lower than those of the control group. However, no significant differences were detected between 3mg/kg group and control. Alanine aminotransferase (34), albumin (35) and RBC number (36) are all important indicators of the safety of a treatment., Melatonin treatment did not significantly altered pathophysiological states of lambs, As a result, melatonin treatment will not affect the normal growth of lambs.

The growth hormone, estradiol and testosterone in lambs with melatonin implantation were significantly increased compared to the controls. These hormones are often associated with muscle growth and body structural formations (37, 38). The effect of melatonin on the increased plasma growth hormone (GH) concentration was reported in Japanese quails (39). Melatonin is reported to reduce androgen level in male Siberian hamsters (40). These results suggest that melatonin can affect hormonal levels in animals. But, the observation is different with our findings and this may relate to the species specific. Melatonin is a potent antioxidant (41). As we observed in the study that it reduced the level of malondialdehyde and increased the activity of SOD. These actions of melatonin may prevent the muscle degradation under oxidative stress of lambs after weaning (42). Most importantly, it was observed that melatonin treatment increased the cross-sectional area of muscle fiber and adipose tissue cells. This is the direct evidence to show how melatonin improved the growth performance of lambs. Genetic information showed that the differentially expressed genes in muscle and adipose tissues are enriched in many functional pathways after melatonin treatment. KEGG analysis found that the most significant differentially expressed genes in muscle and adipose tissues impacted by melatonin was Apoptosis pathway. Apoptosis, which removes aged and damaged cells during development and protects cells from external interference and maintains homeostasis (43). Several studies have reported the effects of melatonin on apoptosis (44, 45). It is possible that the effects of melatonin on muscle growth and development might involve in gene expression of apoptotic signaling pathway.

The muscle and fat transcriptome transcription analysis further demonstrated that melatonin regulated the Apoptosis pathway function. Sixteen genes in Apoptosis pathway were selected for expression verification. Three genes showed the same expression pattern in muscle tissue and adipose tissue with melatonin treatment. These included CytC, PARP1 and IL1-β. CytC is an indispensable element of the electron transport chain in mitochondrial for energy metabolism (46). PARP1 gene is the hub of intracellular inflammatory response, oxidative stress and metabolic response (47). IL1-β is one of the important factors in the inflammatory regulatory network, initiating and controlling the inflammatory response (48). CytC, PARP1 and IL1-β are key genes to maintain metabolism and immunity. These results provide further evidence that melatonin may affect the growth and development of lambs by regulating the expression of these genes in the Apoptosis pathway. In addition to these internal pathways, some external pathways may also participate in the growth performance of lambs. This includes the intestinal microbiota of the lambs. It is well known that these microbiota affect the nutritional and physiological processes of hosts (49). The gastrointestinal tract of mammals inhabits trillions of microorganisms, and different types of intestinal microorganisms maintain a relatively stable number to maintain nutrient absorption (50). It has been reported that melatonin regulates the distribution and metabolism of intestinal microorganisms (51, 52). The crosstalk between the microbiota and the tissues of the host will influence the growth and development status of the host (53). It has been showed that melatonin treatment increased the relative abundance of families Prevotellaceae, Muribaculaceae while it reduced the relative abundance of Succinivibrionaceae, Veillonellaceae in ruminal of lactating cows (54). In this study, it was found that melatonin also affected rumen microflora of lambs. PICRUSt analysis showed that melatonin mainly affected the Cardiovascular disease, Excretory system and Signaling molecules and interaction pathways related to the microbiota. These pathways significantly affect the body health and nutrient absorption of lambs. This suggests that melatonin may also enhance nutrient absorption and promote muscle and fat development in lambs by partially influencing gut microbes.

In conclusion, in this study, we first reported that melatonin implantation im-proved the growth rate of lambs by increasing the sizes of their muscle and fat cells. The potential mechanisms are multiples. These include melatonin increases the growth hormone and androgen secretion, reduces the oxidative stress, enriches the expressions of genes in Apoptosis pathway, such as CytC, PARP1 and IL1-β genes which are the key genes to maintain metabolism and immunity. Finally, melatonin regulates the intestinal microbiota to target their Cardiovascular disease, Excretory system and Signaling molecules and interaction pathways.

**Figure 12:**
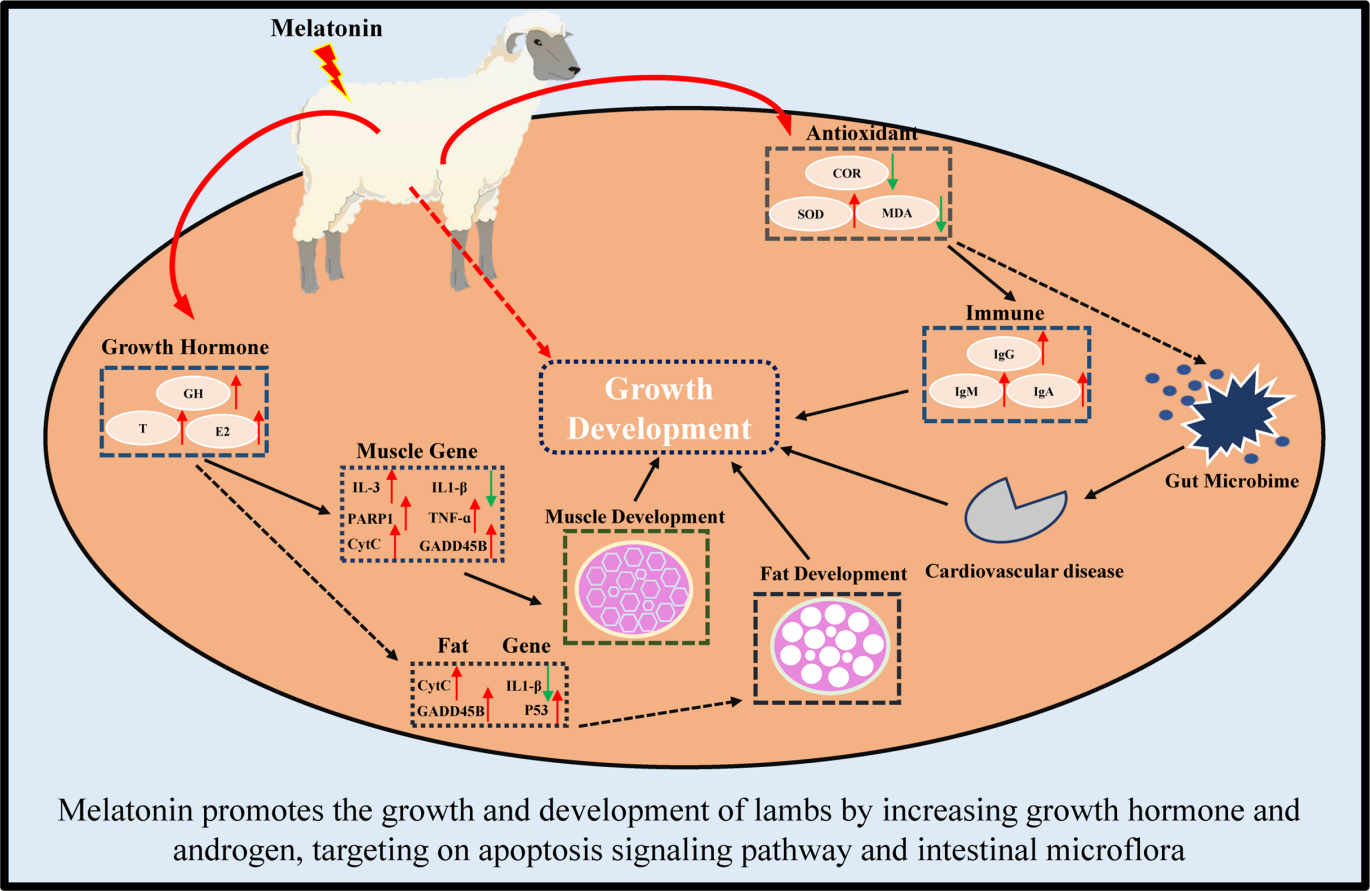
Schematic diagram of melatonin promoting growth and development of lambs.

In conclusion, melatonin implantation improved the growth rate of lambs by increasing the sizes of their muscle and fat cells. The underlying mechanisms are illustrated in **Figure 12**. Melatonin increases the growth hormone and testosterone secretion, reduces the oxi-dative stress in lambs. Melatonin enriches the expressions of genes in Apoptosis pathway, especially in CytC, PARP1 and IL1-β genes. Melatonin regulates the intestinal microbiota to target their Cardiovascular disease, Excretory system and Signaling molecules and interaction pathways. These findings provide novel information for the application of melatonin to improve the production of sheep husbandry in the future.

## Data availability statement

The 16S rRNA microbial sequencing data and Transcriptome sequencing analysis data have been successfully submitted to the National Center for Biotechnology Information. SRA data: PRJNA865106 and PRJNA866689.

## Ethics statement

The animal study was reviewed and approved by Animal Welfare and Experimental Animal Ethics Committee of China Agricultural University (No:AW01502202-1-2). Written informed consent was obtained from the owners for the participation of their animals in this study.

## Author contributions

WM and HW designed the experiments and prepared the original manuscript. GDL and LY performed muscle and fat samples were collected and transcriptome sequencing analysis. LW, MZ, and SG collected and analyzed blood samples. SX and XG are responsible for feeding and management of lamps. FL participates in experimental design and provides technical services. PJ participated in the data analysis of physiological and biochemical indicators. AW participates in experimental design and editing the manuscript. GSL participates in experimental design, supervised the project and revised the article. All authors contributed to the article and approved the submitted version.

## Funding

National Key Research and development Program (2021YFD1300903), the Science and Technology Major Project of Inner Mongolia (2021ZD0023-1); Key Research and Development Project of Hainan Province (ZDYF2021XDNY174); National Transgenic Key Project of the Ministry of Agriculture of China(2018ZX0800801B).

## Acknowledgments

This research thank for National Key Research and development Program (2021YFD1300903), the Science and Technology Major Project of Inner Mongolia (2021ZD0023-1); Key Research and Development Project of Hainan Province (ZDYF2021XDNY174); National Transgenic Key Project of the Ministry of Agriculture of China(2018ZX0800801B).

## Conflict of interest

Authors SX, XG, and FL were employed by Inner Mongolia Golden Grassland Ecological Technology Group Co., LTD., China.

The remaining authors declare that the research was conducted in the absence of any commercial or financial relationships that could be construed as a potential conflict of interest.

## Publisher’s note

All claims expressed in this article are solely those of the authors and do not necessarily represent those of their affiliated organizations, or those of the publisher, the editors and the reviewers. Any product that may be evaluated in this article, or claim that may be made by its manufacturer, is not guaranteed or endorsed by the publisher.
